# Advances in the therapeutic applications of dichloroacetate as a metabolic regulator: A review

**DOI:** 10.1097/MD.0000000000044295

**Published:** 2025-09-05

**Authors:** Xiaohuan Wu, Min Shang, Meichuan Li, Yujuan Liu, Han Hu, Ping Zhang, Qiuyi He, Shide Lin

**Affiliations:** a Department of Infectious Diseases, Affiliated Hospital of Zunyi Medical University, Zunyi, Guizhou, China.

**Keywords:** adverse effect, dichloroacetate, glycolysis, mitochondria, therapeutic applications

## Abstract

Dichloroacetate (DCA), as a pan-inhibitor of pyruvate dehydrogenase kinase, plays a crucial role in energy metabolism and mitochondrial function. DCA decreases lactic acid synthesis, enhances mitochondrial oxidative phosphorylation, and regulates aerobic glycolysis. During the last decade, more and more studies have found that disorders of energy metabolism and mitochondrial dysfunction play a pivotal role in the development and progression of various diseases, and the role of DCA in cancer, metabolic diseases, and inflammatory diseases has been extensively explored in both basic and clinical studies. In this review, we summarize advances in the therapeutic applications of DCA as a metabolic regulator.

## 1. Introduction

Dichloroacetate (DCA) was first discovered as a metabolic modulator in 1970 to reduce blood glucose levels.^[1]^ It has been utilized in the treatment of lactic acidosis and congenital mitochondrial disease in children.^[2,3]^ During the last decade, numerous studies have demonstrated that energy metabolism disorders and mitochondrial dysfunction contribute significantly to the pathogenesis of various diseases. The effects of DCA on cancer, metabolic diseases, and inflammatory diseases have been extensively investigated. Here, we summarize advances in the role of DCA as a metabolic regulator in the treatment of disease.

## 2. The pharmacological effects of DCA

**Figure 1.**
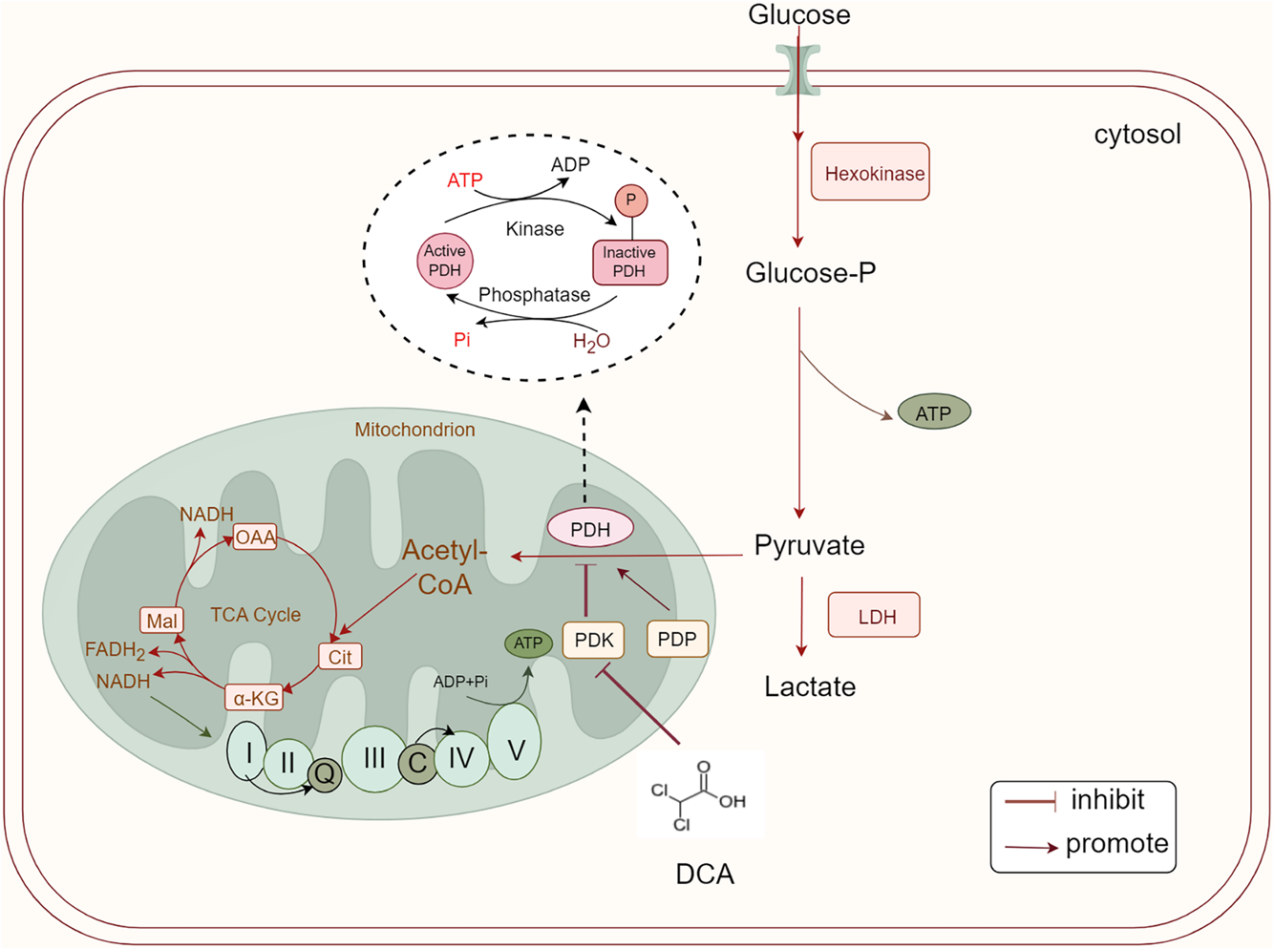
DCA’s pharmacological mechanisms. When PDH is phosphorylated by PDK, it is inhibited, but when PDH is dephosphorylated by PDP, it becomes active. PDK is inhibited by DCA, thus causing PDH to be activated. DCA = dichloroacetate, PDH = pyruvate dehydrogenase, PDK = pyruvate dehydrogenase kinase, PDP = pyruvate dehydrogenase phosphatase.

The key pharmacological action of DCA is the regulation of the mitochondrial pyruvate dehydrogenase (PDH) complex (PDC), which links glycolysis to the tricarboxylic acid cycle and oxidative phosphorylation (OXPHOS). The activity of PDC is regulated by the phosphorylation and dephosphorylation of 3 serine residues (Ser293, Ser300, and Ser232) on the PDH α subunit. Phosphorylation of PDH by pyruvate dehydrogenase kinase (PDK) leads to inactivation of the PDC, whereas dephosphorylation of PDH by pyruvate dehydrogenase phosphatase reactivates PDC.^[4]^ DCA improves cellular energy metabolism by inhibiting PDK, keeping PDC in an active state and promoting the aerobic oxidation of pyruvate (Fig. 1).

There are 4 subtypes of PDK in mammalian cells, PDK1 to PDK4, each of which has a unique affinity. The expression of the PDK subtype is tissue-specific. Under normal physiological conditions, PDK1 and PDK2 are ubiquitously expressed in all tissues, while PDK3 is only found in the testis, lung, kidney and brain, and PDK4 is only found in muscle-derived tissues and brown fat.^[5]^ DCA has high bioavailability in vivo and can stimulate PDC within minutes.^[6]^

## 3. The metabolism of DCA

**Figure 2.**
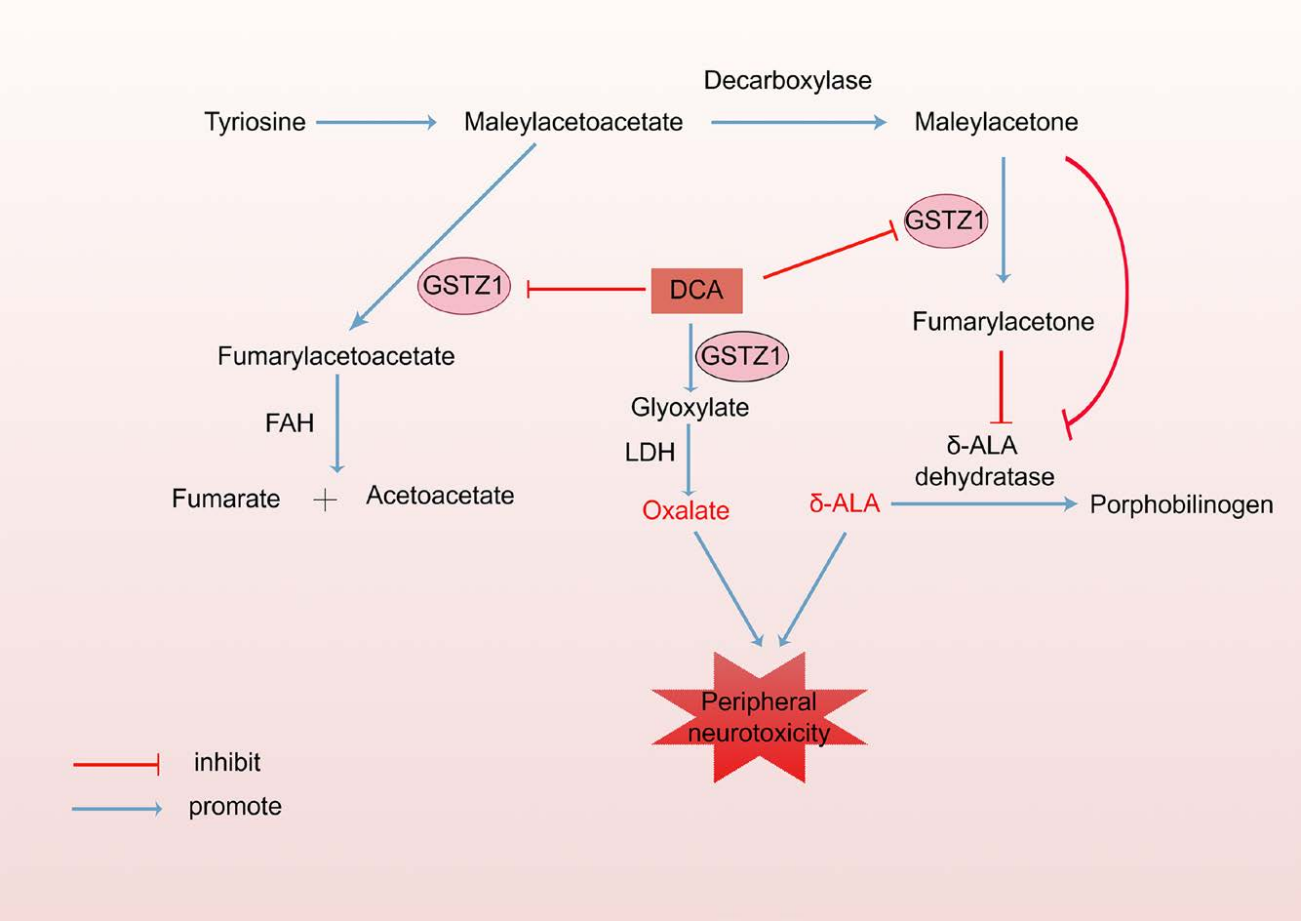
DCA metabolism and the generation of neurotoxic compounds. DCA = dichloroacetate, FAH = fumarylacetoacetate hydrolase, GSTZ1 = glutathione transferase zeta 1, LDH = lactate dehydrogenase, δ-ALA = δ-aminolevulinic acid.

The earliest human pharmacokinetic research on DCA was conducted in the 1980s and demonstrated that the elimination of DCA significantly decreased following repeated administrations.^[7]^ Glutathione transferase zeta-1 (GSTZ1) catalyzes DCA to glyoxylic acid, which is subsequently converted to oxalate by lactate dehydrogenase (Fig. 2). DCA can inactivate GSTZ1, resulting in a self-inhibition of its metabolism.^[8,9]^ GSTZ1 is predominantly distributed in hepatocytes and proximal renal tubule cells. Research has demonstrated that GSTZ1 expression and activity in the liver are higher than in other tissues after a single dosage of DCA in rats, indicating that DCA metabolism primarily occurs in the liver.^[10]^

DCA metabolism is influenced by age, with DCA clearance being higher in children compared to that in adults. The underlying mechanism remains unclear, and it may be associated with the retention of greater amounts of GSTZ1 in children following multiple DCA administrations.^[10]^

Furthermore, the metabolism of DCA was found to be influenced by the genotype of GSTZ1. Based on 3 prevalent single nucleotide polymorphisms in the GSTZ1 coding area, 5 haplotypes can be identified: EGT (GSTZ1C, 70%–80%), KGT (GSTZ1B, 15%–25%), EGM (GSTZ1D, 5%–10%), KRT (GSTZ1A, 1%–10%), and KGM (GSTZ1F, <1%). The presence of an EGT allele is associated with rapid DCA metabolism and fewer adverse events.^[11]^ In a phase II clinical trial of 6 patients with DCA treatment (average dosage of 25 mg/kg/day) for 12 weeks, the patients without the EGT allele experienced more severe neuropathy compared to those with the allele.^[12]^

The most prevalent and severe adverse event associated with long-term DCA administration is reversible peripheral neuropathy.^[13–15]^ Accumulation of the metabolite oxalate can lead to peripheral neurotoxicity because its metabolism depletes thiamin.^[16]^ Consequently, thiamin supplementation may ameliorate or prevent the onset of peripheral neuropathy.^[17]^

In addition, the accumulation of the neurotoxic substance δ-aminolaevulinic acid (δ-ALA) also contributes to the development of peripheral neuropathy.^[11]^ The intermediates of tyrosine catabolism, maleylacetoacetate and maleylacetone (MA), are physiological substrates of GSTZ1. GSTZ1 converts maleylacetoacetate and MA to fumarylacetoacetate and fumarylacetone. In the presence of fumarylacetoacetate hydrolase, fumarylacetoacetate is converted into fumarate and acetoacetate, both of which are nontoxic. The accumulation of MA and fumarylacetone, resulting from GSTZ1 inhibition during the prolonged administration of DCA, prevents ALA dehydratase from converting δ-ALA to porphobilinogen, thereby resulting in δ-ALA accumulation (Fig. 2).

## 4. Therapeutic applications of DCA

### 4.1. Diabetes mellitus

Over 50 years ago, it was found that DCA could reduce blood glucose levels in diabetic rats, and its mechanism may be related to the enhancement of glucose oxidation in peripheral tissues and the inhibition of gluconeogenesis.^[1,18,19]^ In clinical trials, oral administration of DCA (3–4 g) in diabetic patients for 6 to 7 days significantly reduced fasting blood glucose levels, but it has not been widely used in the treatment of diabetes because of the risk of peripheral neuropathy associated with long-term application.^[20]^

Recent studies have shown that DCA exerts beneficial effects on diabetes-related complications. In a cellular model of proliferative retinopathy, DCA had a strong anti-epithelial-mesenchymal transition effect.^[21]^ Further animal studies have shown that DCA attenuates retinal degeneration in rd10 mice.^[22]^ In addition, DCA attenuated epithelial-mesenchymal transition and oxidative stress in a cellular model of diabetic cataracts. Animal studies showed that DCA significantly reduced lens clouding in diabetic rats and dose-dependently improved diabetic cataract formation and progression.^[23]^ All these studies suggest that DCA has a protective effect against diabetes-related ophthalmic diseases.

Furthermore, diabetic cardiomyopathy is associated with reduced PDH levels due to elevated PDK4. In diabetic rats, DCA treatment increased PDH activity, restored myocardial substrate-selective homeostasis, and improved cardiac function.^[24]^ DCA was also found to improve ovarian and endometrial damage and improve ovarian reserve function induced by diabetic rat models.^[25]^

Although DCA has shown beneficial effects in diabetes and diabetes-related complications, clinical trials are lacking. Future individualized dosing based on age and GSTZ1 genotype is needed to validate its role in diabetic patients.

### 4.2. Lactic acidosis

DCA has been widely used in lactic acidosis due to inherited mitochondrial diseases. Several randomized controlled clinical trials and open-label studies have demonstrated that long-term oral administration of DCA at 25 mg/kg/day is well tolerated and maintains normal blood lactate levels in young children with various congenital mitochondrial diseases.^[3,26,27]^ In a recent case report of severe lactic acidosis in a neonate with mitochondrial methionyl-tRNA formyltransferase deficiency, DCA treatment (30 mg/kg/day) normalized lactate levels, reversed cardiomyopathy, and resulted in long-term stabilization of clinical status.^[28]^

However, in patients with mitochondrial myopathy, encephalopathy, lactic acidosis, and stroke-like episodes, DCA induced peripheral neuropathy after 3 years of treatment with DCA at a dose of 25 mg/kg/day.^[29]^ Notably, these patients were older (mean age at enrollment was 30 years). DCA metabolism slows down with age, and DCA-induced GSTZ1 inactivation is 25 to 30 times higher in adults than in children,^[30]^ which increases the risk of peripheral neuropathy in adults. In another study of patients with congenital lactic acidosis receiving longer-term treatment with DCA, 8 patients (most of whom were younger than 10 years of age) were followed up and received DCA for up to 9.7 to 16.5 years without worsening peripheral neuropathy.^[31]^ A possible explanation for this result is that these patients were younger and were rapid metabolizers of DCA.

In summary, DCA is usually safe and well tolerated in the long-term treatment of young children with congenital lactic acidosis of all causes, but there are difficulties in the treatment of adults with primary mitochondrial disease.

In patients with acquired lactic acidosis, DCA is effective in reducing blood lactate levels and stabilizing acid-base balance.^[2,32,33]^ Unfortunately, it has no effect on hemodynamics and survival, and the final prognosis depends on the underlying disease.^[34]^ Animal studies have indicated that DCA can pass the placental barrier into the fetal circulation and lower fetal plasma lactate levels,^[35]^ suggesting DCA may have possible clinical application value in the treatment of intrapartum lactic acidosis.

### 4.3. Cancer

Most cancers are characterized by increased aerobic glycolysis. Cancer cells utilize glycolysis to generate energy even when oxygen is present, which is known as the Warburg effect.^[36]^ This disrupted metabolic process in cancer cells is associated with mitochondrial dysfunction caused by the inhibition of PDC. Overexpression of PDK in cancer cells is induced by a variety of factors, such as hypoxia-inducible factor 1-α (HIF-1α) and peroxisome proliferator-activated receptor α.^[4]^

Targeting PDK to reverse the metabolic phenotype of malignant tumors is currently a research hotspot in tumor therapy. Numerous studies have assessed the possible roles of DCA in cancer. In studies with animal models of different tumors or with different cancer cell lines, promising results have been obtained. The mechanism discovered in most studies is related to the inhibition of mitochondrial PDK by DCA, which enhances the shift from glycolysis to OXPHOS and promotes apoptosis of malignant cells.^[37,38]^ Additionally, several other mechanisms may also be involved. Upregulation of HIF-1α in cancer leads to increased expression of vascular endothelial growth factor and other angiogenic molecules, promoting tumor proliferation and metastasis^[39]^; and it has been found that DCA inhibited HIF-1α expression, and suppressed tumor angiogenesis and proliferation.^[40]^ In addition, DCA inhibits β-oxidation of fatty acids, leading to reduced nucleotide synthesis and cancer cell replication.^[41,42]^ Lactic acid accumulation in the tumor microenvironment can inhibit T-cell proliferation and cytokine signaling, and DCA has been found to improve T-cell function and ameliorate immunosuppression by reducing lactate production.^[43,44]^ Thus, DCA seems to enhance the immune system for an antitumor response.

Recently, many in vitro and in vivo studies found that DCA can increase cancer cell susceptibility to chemoradiotherapy and reduce the side effects caused by chemotherapy.^[37,45–47]^ 5-Fluorouracil and a new derivative of DCA can improve the treatment efficiency and reduce the side effects caused by 5-fluorouracil.^[48]^ DCA combined with doxorubicin also protects against the cardiotoxicity caused by doxorubicin.^[49]^ The combination of DCA and metformin inhibited the proliferation of liver cancer cells, and may be beneficial for those with end-stage liver cancer.^[50]^

However, the results from the limited clinical trials are inconsistent regarding the antitumor effects and survival benefits. In a phase II clinical study of DCA combined with chemoradiotherapy for patients with advanced head and neck squamous cell carcinoma, DCA reduced pyruvate and lactate but did not improve survival.^[51]^ In another open-label phase II trial, DCA therapy showed no clinical benefit in 6 patients with advanced non-small cell lung cancer and 1 patient with breast cancer, side effects occurred, and 2 patients died after receiving therapy. The trial was ended early for safety reasons, but it is unclear whether the occurrence of adverse events was associated with DCA.^[52]^ In another open-label, phase I trial of 23 persons with solid tumors treated with DCA, it was found that 8 of 17 evaluable patients had stable disease, however, the majority were unable to continue treatment.^[53]^

In contrast, Michelakis et al conducted a clinical study, in which oral DCA (12.5–25 mg/kg, twice a day) was given to 5 glioblastoma patients for 15 months, and they found 3 patients experienced tumor regression without peripheral neuropathy.^[40]^ In another study, 15 adult patients with recurrent malignant brain tumors were administered with a dosage of 12.5 mg/kg twice a day for rapid DCA metabolizers, and clinical and radiological evidence of disease stabilization was found in 8 evaluable patients after 4 weeks of treatment, and no signs of toxicity were found.^[54]^ In a phase II open-label study, Tian et al stratified DCA dosage according to GSTZ1 genotype among 7 myeloma patients, and they found 1 patient achieved complete remission and 2 patients achieved remission.^[12]^ In a case report of a patient with relapsed non-Hodgkin lymphoma, complete clinical remission was also achieved after DCA treatment.^[55]^ Furthermore, case reports have demonstrated the clinical benefits of DCA in patients with metastatic melanoma,^[56]^ colon cancer,^[57]^ stomach cancer,^[58]^ and cholangiocarcinoma.^[59]^

DCA’s poor binding affinity for PDK means that it is only effective if administered at high dosages for a long period. However, the peripheral neuropathy caused by prolonged and high-dose administration has limited its clinical application.^[27]^ The development of more efficient PDK inhibitors is an effective way to address this clinical limitation. Over the past decade, scientists have developed a number of DCA derivatives that have improved the efficacy of cancer treatment,^[60–62]^ but there is still a lack of efficient PDK inhibitors. Recently, researchers have developed a new potent PDK inhibitor, a conjugated compound of DCA with arsenic precursors, which shrank 90% of tumors under highly loaded nanoparticle administration without any significant toxicity.^[63]^ It also suggests that the application of targeted delivery technology may be an effective way to augment DCA for the treatment of cancer by releasing high doses of cytotoxic substances into cancer cells without damaging normal cells.^[61,63]^

Secondly, the GSTZ1 genotype-based dosing regimen was found to be safer for long-term administration of DCA in clinical studies,^[12,54]^ but the number of cases was small and it should be included in future chronic dosing trials.

These studies provide exciting opportunities for future clinical applications of DCA in cancer, and more studies are needed in the future to validate the efficacy and safety of DCA and its derivatives in cancer treatment.

### 4.4. Pulmonary arterial hypertension

Pulmonary arterial hypertension (PAH) is a rare cardiopulmonary condition characterized by severe remodeling of the peripheral pulmonary arteriole, which causes a progressive rise in pulmonary vascular resistance and, ultimately, right heart failure. Approximately 40% of individuals die within 5 years of being diagnosed with right heart failure.^[64,65]^ Thus, exploring novel therapeutic targets is necessary.

The hallmark feature of PAH is aerobic glycolysis. Increased glycolysis and inhibited mitochondrial glucose oxidation have been observed in PAH patients and PAH animal models, which are similar to tumor cells, resulting in excessive proliferation of pulmonary vascular cells, apoptotic resistance, excessive migration, and dysregulation of cell energy, ultimately leading to thickening and remodeling of pulmonary arteries.^[66–68]^ Mitochondrial dysfunction may be an important pathogenesis of PAH.^[67,69]^ Pulmonary vascular remodeling and contraction are primarily attributed to the dysfunction of the pulmonary arterial smooth muscle cell (PASMC).

In experimental PAH, DCA has been reported to improve cardiac function and survival by reversing aerobic glycolysis and inducing caspase-mediated PASMC apoptosis.^[70,71]^ Furthermore, DCA improves bioenergetic and right ventricular function in patients with right ventricular hypertrophy by inhibiting FOXO1-induced PDK4 overexpression and restoring glucose oxidation.^[72]^ A recent study showed that in a rat model of PAH, a new synthetic drug, Fasudil DCA, improved hypoxia-induced PASMC dysfunction by blocking the Ca^2+^/calmodulin-dependent kinase and Rho kinase signaling pathways while also maintaining mitochondrial homeostasis, thereby alleviating the PAH.^[73]^ HIF-1α is a known transcription regulator of PDK.^[74]^ In monocrotaline-induced Sprague-Dawley rats, HIF-1α increased the expression of PDK and activated connective tissue growth factor and TGF-β1, resulting in the proliferation of right ventricular fibroblasts. DCA reduces phosphorylated PDH expression, increases mitochondrial oxidation, inactivates HIF-1α, attenuates right ventricular fibrosis and hypertrophy, and thus improves right ventricular function.^[75]^

In addition, Michelakis et al found that DCA administration activated PDH and increased mitochondrial respiration, resulting in lower mean pulmonary artery pressure and pulmonary vascular resistance in patients with idiopathic PAH in a 4-month clinical study.^[67]^ This is the first human trial of DCA in patients with idiopathic PAH, which suggests that PDK is an efficient target for PAH therapy. DCA may become a promising medicine for treating PAH in the future.

### 4.5. Sepsis

Sepsis is a severe, life-threatening disease with at least 19 million cases reported worldwide annually and a high mortality rate.^[76]^ Lactic acidosis is a hallmark of sepsis and septic shock, and increased lactate concentration is related to a worse outcome. The accumulation of lactic acid is caused by insufficient oxygen supply, impaired tissue oxygen uptake, decreased lactate scavenging, and increased glycolysis.^[77]^ DCA may be a potentially effective treatment for sepsis and septic shock because of its ability to effectively lower blood lactate levels.

Unfortunately, clinical trials have shown that although DCA can effectively lower blood lactate concentration, it cannot increase survival rates.^[34,78]^ Stacpoole et al conducted a randomized controlled study of DCA and placebo in 252 adult patients with lactic acidosis. In more than half of these patients, sepsis was the cause of lactic acidosis. Among the 126 patients with DCA, 83 (66%) exhibited a 20% or greater decrease in arterial blood lactate concentration, but it was not associated with increased survival. The possible explanation for this result is that the patient’s condition is too serious and the overall survival time may be unaffected by additional changes in acid-base status.^[34]^ Interestingly, in a case report of septic neonates with severe lactic acidosis, DCA decreased lactic acid to normal after early treatment and improved the clinical status.^[79]^ This suggests that applying DCA early in the course of the disease may be beneficial for septic patients.

Research on the role of DCA in sepsis is ongoing. Recent animal studies indicate that DCA can reverse immune paralysis and enhance survival in mice with sepsis induced by cecal ligation and puncture. The immune-enhancing effects of DCA were evidenced by improved peritoneal bacterial clearance in the absence of antibiotics.^[80]^ Concurrently, an increase in β-catenin was observed, indicating that DCA may facilitate cell and organ regeneration. Further studies have shown that DCA improved immunoparalysis in septic vital organs, reversed hepatocellular metabolic disorders and mitochondrial dysfunction, restored hepatic fuel metabolism, and ameliorated systemic energy imbalance by inhibiting PDK, stimulating mitochondrial OXPHOS metabolism.^[81,82]^ In addition, the study found that DCA combined with antibiotics extended the lifespan of flies with sepsis.^[83]^ Mitochondrial dysfunction due to increased PDK4 has been observed in vitro models of sepsis-induced cardiomyopathy, and DCA inhibits PDK4 to reduce lipid accumulation and calcium overload, reduce lactic acid production, and improve mitochondrial function.^[84]^ Recent in vitro and in vivo research has shown that DCA decreases sepsis-induced acute kidney injury.^[85]^

Recent studies have demonstrated the beneficial effects of DCA in sepsis and related complications. Considering the short duration of sepsis, DCA treatment is limited to a single dose or a short course without the side effect limitations of its long-term use, which brings more opportunities for the clinical use of DCA in sepsis.

### 4.6. Ischemia-reperfusion injury

Ischemia-reperfusion injury (I/RI) refers to tissue damage and metabolic disturbances that occur after the restoration of blood flow to partially ischemic tissue. Common diseases associated with I/RI include trauma, hemorrhagic shock, ischemic stroke, myocardial infarction, acute kidney injury, and cardiac arrest.^[86]^

Hemorrhagic shock is the most prevalent preventable cause of death in trauma.^[87]^ Numerous studies have demonstrated that mitochondrial dysfunction is a significant mechanism underlying multiple organ failure in hemorrhagic shock.^[88,89]^ Hemorrhagic shock induces hypoxia, which subsequently increases PDK activity, thereby inhibiting OXPHOS.^[90,91]^ Research indicates that DCA enhances mitochondrial function and prolongs survival time in rats experiencing hemorrhagic shock without fluid resuscitation; however, it does not reduce serum lactate concentration or extend survival time during prolonged and severe hemorrhagic shock.^[87,92]^

Recent research has shown that the level of PDK4 in diabetic mice dramatically increases following I/RI. Furthermore, DCA has been shown to mitigate apoptosis after I/RI by decreasing the expression of PDK4 and the phosphorylation levels of PDHE1α. This process also alleviates oxidative stress and reduces the production of inflammatory factors, including TNF-α, IL-6, and IL-1β.^[93]^ In addition, DCA can also reduce the infarct size caused by I/RI, improve myocyte systolic dysfunction, induce intracellular Ca^2+^ signal transduction, and reduce reactive oxygen species (ROS) production. The protective effect of DCA on I/RI myocardium is not only reflected in regulating cardiometabolism but also due to its activation of the AMP-activated protein kinase signaling pathway.^[94]^

Oh et al found that in I/RI-induced acute kidney injury, succinic acid accumulates and is oxidized during reperfusion, which leads to excessive ROS production and severe kidney injury. Inhibition of PDK4 can reduce succinic acid accumulation and inhibit ROS-induced nephrotoxicity.^[95]^ Animal model studies have demonstrated that DCA inhibits PDK, increases PDH activity, and enhances hemodynamics and prognosis following cardiac arrest.^[96,97]^ Enhanced PDH activity by DCA alleviated cardiac systolic dysfunction after ischemia-induced ventricular fibrillation.^[98]^ These studies suggest that single-dose DCA may be valuable in acute critical situations and avoid peripheral neurotoxicity associated with long-term use.

In addition, it has been demonstrated that DCA provides vascular protection in ischemic stroke. PDH activity was reduced during I/RI^[99]^ and DCA increased PDH activity and promoted brain regeneration after cerebral ischemia. In transient cerebral ischemia animal models, after DCA administration, neuronal death and oxidative stress were reduced.^[100]^ Endothelial progenitor cells have been shown to promote angiogenesis and angiopoiesis,^[101]^ and long-term DCA improves cognitive function and reduces cerebral infarction size in rats with vascular dementia, partly due to improved endothelial progenitor cell function.^[102]^

### 4.7. Ulcerative colitis

Ulcerative colitis (UC) is a chronic inflammatory colon disease that causes erosion, ulceration, and bleeding on the mucosal surface of the colon. It is the most common inflammatory bowel disease all over the world. Unfortunately, UC is incurable and prone to recurrence and is closely related to colon cancer.^[103,104]^ However, the majority of current therapies are focused on controlling inflammation and clinical symptoms (such as 5-aminosalicylic acid and corticosteroids), and the search for new therapeutic agents is urgent.

Recent studies have shown that in a mouse UC model, DCA treatment successfully reversed the harmful inflammatory response by suppressing the expression of nuclear factor kappa-B and nucleotide-binding oligomerization domain-like receptor protein 3, cleaving caspase-1 and reducing IL-1β levels.^[104]^ Therefore, DCA is considered to be a novel and promising drug candidate for UC management.

### 4.8. Dyslipidemia and atherosclerosis

Back in the 1970s, researchers found that DCA significantly reduced plasma cholesterol and triglyceride levels in patients with hyperlipidemia.^[20]^ Atherosclerosis induced by dyslipidemia is related to high morbidity and mortality. Elevated levels of low-density lipoprotein in the blood are a major risk factor for atherosclerosis and a major cause of death.^[105]^ Plasma cholesterol levels are primarily controlled by the low-density lipoprotein receptor, which mediates the endocytosis of cholesterol-rich low-density lipoprotein.^[106]^ The specific mechanism by which DCA affects cholesterol remains unclear. Previous research has indicated that DCA is a noncompetitive inhibitor of 3-hydroxy-3-methylglutaryl coenzyme A reductase,^[78]^ and 3-hydroxy-3-methylglutaryl coenzyme A reductase is the rate-limiting enzyme for cholesterol production,^[107]^ which explains its mechanism in lowering cholesterol. In addition, Khan et al found that DCA promotes ERK5/MEF2 pathway activation by increasing OXPHOS, leading to increased low-density lipoprotein receptor expression and decreased plasma cholesterol level.^[108]^

In addition, DCA was found to reduce triglyceride levels in isolated rat hepatocytes by decreasing fatty acid production and promoting fatty acid oxidation.^[109]^ Recent animal experiment has shown that DCA could reduce triglyceride levels and alleviate the progression of nonalcoholic fatty liver disease in mice fed a high-fat diet. The mechanism may be related to its activation of PDH. However, hepatocyte ballooning was observed in mice fed a normal diet.^[110]^

In a mouse model, DCA stimulated AMP-activated protein kinase-mediated fibroblast growth factor 21 mRNA expression and increased the activation of brown adipose tissue, thereby preventing atherosclerosis.^[111]^ DCA targeting the PDK/PDH axis has been found in animal trials to skew the immune system, suppress nucleotide-binding oligomerization domain-like receptor protein 3 inflammasome activation, and limit IL-1β release in macrophages in plaques, leading to plaque stability and reduced vascular inflammation.^[112]^

### 4.9. Other diseases

It was found that DCA stimulated PDH activity, reduced brain lactic acid concentration, significantly increased survival, and improved motor function in mice with Huntington disease.^[113]^ A recent study found that glycolysis is increased in patients with endometriosis and that DCA treatment normalizes peritoneal mesothelial cell metabolism, reduces lactic acid secretion, and reduces lesion size.^[114]^ In vitro studies found that in hepatitis C virus (HCV)-infected hepatocytes, the enzymes involved in glycolysis and serine biosynthesis were upregulated. DCA could block PDK activity, reduce the expression of glycolysis and serine synthetase, and reduce the synthesis of nucleotides necessary for HCV replication, thereby inhibiting HCV replication.^[115]^ Animal studies have found that DCA improved liver function and liver histology in drug-induced acute liver injury, which may be attributed to increased glutathione levels.^[116,117]^ In addition, DCA has shown therapeutic potential in osteoarthritis,^[118]^ toxoplasmosis,^[119]^ and dihydrolipoamide dehydrogenase deficiency.^[120]^ The main therapeutic applications of DCA are summarized in Table 1.

**Table 1:** Summary of therapeutic applications of dichloroacetate.

Disease/condition	Mechanism of action	Key findings	Evidence level	References
Diabetes mellitus	Promoted glucose oxidation; reduced gluconeogenesis; inhibited MAPK	Reduced blood glucose; attenuated retinopathy/cataracts; improved cardiac function	Preclinical	^[19,22–24]^
Lactic acidosis	Reduced the lactate level via PDH activation	Normalized blood lactate; safe in children but neuropathy risk in adults; no hemodynamic/survival benefit in acquired lactic acidosis	Clinical	^[3,28,31,34]^
Cancer	Reversed Warburg effect; inhibited HIF-1α expression; inhibited β-oxidation of fatty acids	Enhanced the sensitivity of cancer cells to radiotherapy and chemotherapy; tumor regression in case series; use limited by neuropathy	Mixed*	^[12,40,48,54,55,63]^
Pulmonary arterial hypertension	PDK inhibition; increased glucose oxidation; induced PASMC apoptosis	Improved hemodynamics; reversed vascular remodeling	Mixed*	^[67,73]^
Sepsis	Lowered blood lactate; reversed immunoparalysis; improved mitochondrial function	Reduced lactate without survival benefit in patients; increased survival in septic animal models; mitigated sepsis-associated complications	Mixed*	^[34,80–82,85]^
Ischemia-reperfusion injury	PDH activation; reduced ROS and alleviated oxidative stress; activated AMPK	Reduced infarct size; protected heart/kidney/brain; acute use avoided neuropathy	Preclinical	^[93–95,98,102]^
Ulcerative colitis	Suppressed NF-κB/NLRP3	Reversed inflammation; reduced disease activity index	Preclinical	^[104]^
Dyslipidemia and atherosclerosis	Inhibited HMG-CoA reductase; increased LDLR; promoted fatty acid oxidation; suppressed NLRP3	Lowered cholesterol/triglycerides; prevented atherosclerosis; reduced vascular inflammation	Preclinical	^[108,111,112]^
Other diseases	PDK inhibition	Benefits in Huntington disease, endometriosis, HCV, liver injury, and osteoarthritis	Preclinical	^[113–115,118]^

AMPK = AMP-activated protein kinase, HCV = hepatitis C virus, HIF-1α = hypoxia-inducible factor 1-α, HMG-CoA reductase = 3-hydroxy-3-methylglutaryl coenzyme A reductase, LDLR = low-density lipoprotein receptor, MAPK = mitogen-activated protein kinase, NF-κB = nuclear factor kappa-B, NLRP3 = nucleotide-binding oligomerization domain-like receptor protein 3, PASMC = pulmonary arterial smooth muscle cell, PDH = pyruvate dehydrogenase, PDK = pyruvate dehydrogenase kinase, ROS = reactive oxygen species.

* Mixed: Preclinical studies + clinical trials/case reports.

## 5. The possible strategy to reduce the side effects of DCA

The most prevalent and serious side effect associated with DCA is reversible peripheral neuropathy.^[13]^ DCA metabolism is influenced by age and GSTZ1 genotype; therefore, in addition to age considerations in long-term DCA administration, adjusted DCA dosage according to GSTZ1 haplotype may be a possible way to ameliorate peripheral neurotoxicity. In a phase I open-label study with 15 adult patients with recurrent glioma, DCA was administered according to GSTZ1 haplotype, and 8 patients completed at least one 4-week cycle (mean 75.5 days) without dose-limiting toxicity while maintaining stable clinical status. The authors concluded that the initial oral dose for patients with and without the EGT allele is 6.25 mg/kg/12 h and 5 mg/kg/12 h.^[54]^ More studies are needed in the future to determine the safe and effective dosage for various age groups and GSTZ1 genotypes.

Combining thiamin and DCA may represent an alternative approach to reducing oxalate accumulation^[121]^; however, whether this combination can mitigate peripheral neurotoxicity remains a subject of controversy. In a case involving a child with complex I deficiency, reversible peripheral neuropathy was still observed after 20 weeks of treatment with the combination of DCA and thiamin.^[122]^

Finally, the design and development of more potent DCA-based PDK inhibitors or targeted drug delivery using novel delivery systems may be possible strategies to address the side effects of DCA and enhance therapeutic efficacy.^[63]^

## 6. Conclusion and prospect

In recent years, the role of DCA in cancer, various metabolic diseases, and inflammatory diseases has garnered increasing attention. Numerous studies have reported its beneficial effects; however, most of these benefits do not translate into clinical improvement. Additionally, peripheral neuropathy due to prolonged administration of DCA has restricted its clinical application. Further studies are necessary to investigate the clinical outcomes and determine the appropriate therapeutic dose of DCA for various diseases and in populations with different metabolic rates. It is exciting that a single-dose or short-term application regimen may make it possible to extrapolate DCA from animal models to humans, especially in certain acute conditions.

In conclusion, current studies have indicated that DCA is a promising drug as a metabolic modulator. Increasing evidence indicates that disorders of energy metabolism and mitochondrial dysfunction play a crucial role in the development of various diseases, and PDC plays a central role in cellular energy metabolism. Based on the pharmacological role of targeting the PDC/PDK axis, DCA is expected to open up exciting prospects for the treatment of many diseases. However, the complexity of DCA metabolism presents significant challenges to its development and application in clinical practice.

## Author contributions

**Funding acquisition:** Shide Lin.

**Writing – original draft:** Xiaohuan Wu, Shide Lin.

**Writing – review & editing:** Xiaohuan Wu, Min Shang, Meichuan Li, Yujuan Liu, Han Hu, Ping Zhang, Qiuyi He, Shide Lin.
